# Effects of dietary rumen-protected glucose level and taurine supplementation on weight change and oxidative stress state of yaks after transport

**DOI:** 10.3389/fvets.2024.1492747

**Published:** 2024-11-21

**Authors:** Xiaolin Wang, Kaiqiang Zhao, Shoupei Zhao, Jia Zhou, Mingyu Cao, Lianghao Lu, Yuanyuan Chen, Huaming Yang, Bao Zhang, Chong Shao, Yanfei Zhao, Rui Tang, Bai Xue

**Affiliations:** ^1^Animal Nutrition Institute, Sichuan Agricultural University, Chengdu, China; ^2^Chongqing Academy of Animal Sciences, Chongqing, China

**Keywords:** rumen-protected glucose, taurine, yak, transport, stress response

## Abstract

Rumen-protected glucose (RPG) and taurine (TAU) are crucial for the nutrition and physiology of ruminants, enhancing production performance and mitigating negative energy balance. This study aimed to assess the impact of RPG levels and TAU supplementation on the body weight, antioxidant capacity, immune function and stress responses in yaks before and after transport. Thirty-two healthy male yaks, aged 3 years and weighing 172.5 ± 10.2 kg, were randomly assigned to one of four dietary treatments: (1) low RPG and low TAU (LRLT), with 1% RPG and 5 g/d TAU; (2) low RPG and high TAU (LRHT), with 1% RPG and 15 g/d TAU; (3) high RPG and low TAU (HRLT), with 3% RPG and 5 g/d TAU; and (4) high RPG and high TAU (HRHT), with 3% RPG and 15 g/d TAU. The yaks were treated with the corresponding diet for 7 days, then received 9 h of transportation, and finally fed the same diet at their destination for 30 days. The weight was measured before and on days 0 and 30 after transport, and the plasma was collected before and on day 0, 10, and 30 after transport for analysis of biochemical, antioxidant, immune, and stress response indicators. We observed that transport increased plasma concentrations of total cholesterol, total protein, lactate dehydrogenase, creatine kinase, malondialdehyde, cortisol and lipopolysaccharides of yaks among treatments, while decreased their BW and plasma IL-10 concentration. Increasing TAU supplementation reduced weight loss (8.42 vs. 11.9 kg) and weight loss percent (4.83% vs. 6.87%) in yaks after transport. The concentration of MDA in plasma was lower in HRHT than in LRLT at day 0 after transport (*p* = 0.03). The activity of GSH-PX was higher in HRHT than in LRLT at day 10 after transport (*p* = 0.04). Concentrations of IL-10 at day 0 and 10 after transport was higher in HRHT than in LRLT and HRLT (*p* = 0.02, *p* = 0.01, respectively). With the increase of TAU supplementation, concentrations of IL-1β at day 30 after transport (*p* = 0.02), TNF-*α* at day 0 after transport (*p* = 0.02), COR at day 10 (*p* = 0.03) and 30 (*p* = 0.05) after transport as well as LPS at day 0 after transport (*p* = 0.04) decreased. In addition, concentrations of COR at day 0 after transport was lower in LRHT and HRHT than in LRLT (*p* = 0.03). Based on all the results, we demonstrated that increasing TAU supplementation levels reduced post-transport weight loss in yaks, improved their antioxidant capacity and immune function, and alleviated stress responses. Considering the effect of resistance to transport stress and costs, the optimal treatment identified in this study involved a diet containing 1% RPG and supplemented with 15 g/d TAU.

## 1 Introduction

Yak is a vital component of animal husbandry in China’s high-altitude grazing regions, providing herders with high-quality milk, meat, and fur, thus holding significant economic value. Grazing on native pasture is the traditional way of raising yaks. Yet, these pastures experience substantial seasonal fluctuations in forage availability, often leading to considerable stress on the pastures, particularly in winter (1). Typically, due to the intake of nutrients falling below the necessary maintenance levels, the weight of grazing yaks will be reduced by about 25% after a cold season, which extends the feeding cycle of yaks, reduces the slaughter yield, and seriously hinders the sustainable development of the yak industry (2, 3). Off-site fattening has increasingly become the primary feeding method for yaks during the cold season, mitigating the traditional contradiction between the forage availability and livestock demand in grazing areas, while enhancing the growth efficiency and meat quality of yaks, resulting in higher economic returns.

Some herders relocate yaks from high-altitude grazing areas to low-altitude farms for breeding. However, live transportation means yaks are exposed to multiple stressors, including noise, crowding, starvation and high temperature, which can exacerbate the adaptive and defensive responses, causing transport stress and compromising health and welfare in animals (4, 5). It has been reported that feed and water deprivation during transport caused dehydration in the body, weight loss and negative energy balance, thereby damaging organismal metabolism and reducing slaughter value (6). Furthermore, prolonged crowding and standing can increase muscle tension and fatigue, and may also promote the development of respiratory diseases (7, 8). Currently, nutritional regulation is a commonly used measure to alleviate transport stress in animals.

Rumen-protected glucose (RPG) can be released directly from the small intestine into the blood circulation, because its surface is covered with special materials like fatty acids, preventing it from being fermented and degraded in the rumen. Unlike monogastric animals, ruminants have a strong dependency on the endogenous glucose produced by gluconeogenesis, so exogenous glucose intake is relatively low. The RPG supplement can change energy supply path, improve energy utilization efficiency, and alleviate negative energy balance (9–11). Zhang et al. (12) also reported that RPG enhanced the large intestine fermentation function and mucosal immunity of transition dairy cows. Taurine (TAU) is a sulfur-containing amino acid that plays an important role in muscle growth, immune response and energy metabolism (13, 14). However, the TAU synthesized in the liver of most mammals has a limited ability to remove free radicals, while supplementation with exogenous TAU improved the antioxidant enzyme activity of animals and their liver tissues, thereby enhancing antioxidant capacity (15, 16).

Our previous study uncovered that supplementing with RPG and TAU reduced weight loss in yaks during transportation. However, the optimal addition of both supplements to effectively alleviate transport stress in yaks remains unclear. Our study aimed to investigate: (1) the effects of four supplemental levels on the weight of yaks before and after transportation; (2) the changes of plasma antioxidant, immune and stress response indicators before and after transportation; and (3) the impacts of continuous supplementation of RPG and TAU on the above indicators in yaks. We hypothesized that one of the four additive combinations of RPG and TAU could minimize the adverse effects of transportation on growth performance in yaks, and that sustained treatment could improve their antioxidant capacity, immunity and oxidative stress status. This study provides evidence for the use of nutritional intervention to alleviate the stress of yaks during transportation.

## 2 Materials and methods

### 2.1 Animals and experimental design

All animal procedures adhered to the Chinese Guidelines for Animal Welfare, and the study protocols were approved by the Experimental Animal Committee of the Animal Nutrition Institute at Sichuan Agricultural University (Approval no.: SCAUAC2019-36).

The study was conducted from January to March 2023. Thirty-two healthy male yaks, aged 3-years; and weighting 172.5 ± 10.2 kg, were assigned randomly into 4 different treatments: (1) low RPG and low TAU (LRLT), the level of RPG in the diet was 1%, and the addition of TAU was 5 g/d; (2) low RPG and high TAU (LRHT), the level of RPG in the diet was 1%, and the addition of TAU was 15 g/d; (3) high RPG and low TAU (HRLT), the level of RPG in the diet was 3%, and the addition of TAU was 5 g/d; and (4) high RPG and high TAU (HRHT), the level of RPG in the diet was 3%, and the addition of TAU was 15 g/d. Ingredients and chemical composition of diets offered to yaks are shown in Table 1. The RPG and TAU used in our study were purchased from Shanghai Meinong Biotechnology Co., Shanghai, China, and Qingdao Kailide Biotechnology Co., Ltd., Qingdao, China, respectively. Both had active ingredients at 50% and a small intestine release rate of 90%. Each treatment involved eight replicates of one yak each replicate. Yaks were housed individually in pens within a pasture in Hongyuan County, Sichuan Province, and were fed a total mixed ration (TMR) twice a day (0700 and 1900 h) during the trial, with a guaranteed daily feed intake of 105–110%. The feed was pushed at least 10 times daily, and yaks had unrestricted access to fresh water throughout the study period. After 7 days on different diets, the yaks were treated for 9 h of long-distance transport. The yaks were fasted for 12 h prior to transportation and loaded into a semi-trailer measuring (9 m × 2.55 m × 4 m) at 10:00 AM on the day of transport, and then transported for 9 h on expressways until they arrived at the Research Farm of Animal Nutrition Institute, Sichuan Agricultural University (Yaan, China). Fasting and water prohibition during transportation, and no feed and fresh water are also provided to yaks within 1 h after transportation. The yaks were eventually raised here for 30 days during which they were fed the same diet as prior to their transportation.

**Table 1:** Ingredients and chemical composition of diets offered to yaks.

Items	Treatments^1^	
	LR	HR
Ingredient composition, % of DM		
*Leymus chinensis*	27.0	27.0
Straw	35.0	35.0
Corn	15.5	14.7
Wheat bran	5.60	5.00
Soybean meal	9.00	9.40
Rapeseed meal	3.00	3.00
Palm oil	2.00	1.00
Sodium chloride	0.20	0.20
Premix^2^	1.70	1.70
RPG	1.00	3.00
Chemical composition, % of DM		
NEg^3^ (MJ/kg)	5.97	5.97
CP	11.6	11.9
NDF	46.1	45.8
ADF	30.7	30.4
Calcium	0.52	0.50
Available phosphorus	0.36	0.33

^1^LR diet (Low RPG): Both LRLT and LRHT treated yaks were provided with this diet, which contains 1% RPG but has TAU content of 5 g/d (LRLT) and 15 g/d (LRHT) respectively; HR diet (High RPG): Both HRLT and HRHT treated yaks were provided with this diet, which contains 3% RPG but has TAU content of 5 g/d (HRLT) and 15 g/d (HRHT) respectively. ^2^The premix provided the following per kilogram of the diet: vitamin A 4500 IU, vitamin D 1500 IU, vitamin E 200 IU, Co 12 mg, Cu 800 mg, Cl 29.5 g, Zn 1,500 g, Fe 3,000 mg, Mn 1,200 mg, I 25 mg, Se 15 mg. ^3^The net weight gain energy (NEg) is calculated, and the rest is measured.

### 2.2 Determination of growth performance

Yaks were weighed both before transport and on days 0 and 30 post-transport to analyze changes in the body weight (BW) of the experimental yaks. The BW loss percent of yaks during transportation was calculated using the formula: BW loss percent (%) = [(BW before transport – BW after transport)/ BW before transport] × 100%. From day 0 post-transport to day 30, daily feed intake was measured to determine dry matter intake (DMI). The yaks were weighed again on d 30, and the average daily gain (ADG) and feed conversion ratio (FCR) were calculated.

### 2.3 Sample collection

Blood samples were collected using vacuum blood collection tubes from the jugular vein of yaks 3 h after morning feeding before transport and on d 0, 10 and 30 after transport. Blood samples were allowed to stand for 30 min and then centrifuged at 4°C and 3,000 × g for 15 min to prepare plasma. The plasma was stored at-20°C for subsequent analysis of biochemical, antioxidant, immune, and stress response indicators.

### 2.4 Determination of biochemical, antioxidant, immune and stress response indicators in plasma

Plasma biochemical, including total cholesterol (TC), total protein (TP), alanine aminotransferase (ALT), lactate (LAC), triglyceride (TG), glucose (GLU), lactate dehydrogenase (LDH) and creatine kinase (CK) were determined using a fully automatic bio-chemical analyzer (Hitachi 3,100, Olympus Optical, Tokyo, Japan). Plasma antioxidant indexes, including activities of catalase (CAT, A007-1-1), superoxide dismutase (SOD, A001-1-2), and glutathione peroxidase (GSH-PX, A005-1-2), as well as total antioxidant capacity (T-AOC, A015-1-2) and malondialdehyde (MDA, A003-1-2) concentrations, were evaluated with an antioxidant assay kit supplied by the Nanjing Jiancheng Bioengineering Institute (Nanjing, China). Plasma immune indexes, like IL-1β (MB-9650A), IL-4 (MB-4904A), IL-6 (MB-4905A), IL-10 (MB-4931A), TNF-*α* (MB-4838A) concentrations, were determined using an ELISA kit from Jiangsu Enzymebiao Bio-technology Co., Ltd. (Nanjing, China), following the manufacturer’s instructions. For plasma stress response indicators, concentrations of cortisol (COR, EB0062) and lipopolysaccharides (LPS, EU3126) were tested by ELISA kit from FineTest, Wuhan Fine Biotech Co. (Wuhan, China).

### 2.5 Statistical analysis

Before comparing treatments, the normality and variance homogeneity of all data were verified. Growth performance, plasma antioxidant, immune and stress response indicators of yaks were subjected to two-way ANOVA by the SPSS statistical software version 22.0 (IBM SPSS, Chicago, IL, USA) to determine the effects of RPG and TAU supplemental levels on the above indexes. When the interaction between RPG and TAU levels in diets was significant, one-way ANOVA was conducted to compare two levels of TAU supplementation within the same RPG levels and two levels of RPG in the diet within the same TAU supplementation. In addition, we also compared the changes of BW, plasma biochemistry, antioxidant, immune and stress response indicators of yaks before and after transport using ANOVA by the SPSS statistical software version 22.0 (IBM SPSS, Chicago, IL, USA). All results are reported as least squares means ± SEM. The Tukey multiple comparison test was employed to identify statistically significant differences at *p* ≤ 0.05.

## 3 Results

### 3.1 Weight changes in yaks before and after transportation

The effects of dietary RPG level and TAU supplementation on the changes of yak BW before and after transportation are shown in Table 2. There was no significant interaction between RPG and TAU regarding the weight of yaks pre-and post-transport, weight loss as well as weight loss percent (*P* > 0.05). Compared with before transportation, the weight of yaks decreased after transportation (*P* < 0.01). Increasing TAU supplementation reduced weight loss (*p* = 0.05, 11.9 and 8.42 kg in LT and HT, respectively). The weight loss percent also decreased (*p* = 0.04) with the increase of TAU supplementation.

**Table 2:** Changes in BW of yaks^1^ before and after transport under various treatments.

Items	Treatment^2^				SEM	R		T		*P*-value^3^			
	LRLT	LRHT	HRLT	HRHT		LR	HR	LT	HT	S	R	T	R*T
BW before transportation (kg)	170.7	171.8	174.7	173.1	5.34	171.2	173.9	172.7	172.4	< 0.01	0.47	0.93	0.89
BW after transportation (kg)	158.5	162.6	163.2	165.3	5.16	160.6	164.3	160.8	163.9		0.31	0.39	0.62
Weight loss (kg)	12.2	9.06	11.5	7.78	2.44	10.6	9.64	11.9	8.42	–	0.57	0.05	0.25
Weight loss percent (%)	7.20	5.27	6.53	4.39	1.38	6.23	5.47	6.87	4.83	–	0.45	0.04	0.20

^1^Values are least squares means of eight replicate yaks (*n* = 8). ^2^LRLT, the dietary addition of RPG was 1%, and the addition of TAU was 5 g/d; LRHT, the dietary addition of RPG was 1%, and the addition of TAU was 15 g/d; HRLT, the dietary addition of RPG was 3%, and the addition of TAU was 5 g/d; and HRHT, the dietary addition of RPG was 3%, and the addition of TAU was 15 g/d. In the “R” to the right of this line, “LR” refers to LRLT and LRHT, while “HR” refers to HRLT and HRHT; In the “T,” “LT” refers to LRLT and HRLT, while HT refers to LRHT and HRHT. ^3^“S” means the transportation; “R” means the RPG; “T” means the TAU; and “R*T” means the interaction between RPG and TAU.

### 3.2 Growth performance in yaks after transportation

The effects of dietary RPG level and TAU supplementation on yak growth performance from days 0 to 30 post-transportation are presented in Table 3. The level of RPG in the diet and TAU supplementation did not affect final BW, ADG, DMI and FCR in yaks (*P* > 0.05).

**Table 3:** Effects of RPG and TAU levels in the diet on the growth performance of yaks^1^ 0–30 d after transportation.

Items	Treatment^2^				SEM	R		T		*P*-value^3^		
	LRLT	LRHT	HRLT	HRHT		LR	HR	LT	HT	R	T	R*T
Final BW (kg)	187.9	189.4	193.5	193.9	5.39	188.7	193.7	190.7	191.8	0.18	0.80	0.62
ADG (kg)	0.57	0.59	0.63	0.69	0.08	0.58	0.66	0.60	0.64	0.19	0.48	0.51
DMI (kg)	4.34	4.41	4.32	4.45	0.08	4.38	4.39	4.33	4.43	0.86	0.08	0.35
FCR	7.93	8.26	7.51	6.85	0.77	8.10	7.18	7.73	7.55	0.29	0.16	0.69

^1^Values are least squares means of eight replicate yaks (*n* = 8). ^2^LRLT, the dietary addition of RPG was 1%, and the addition of TAU was 5 g/d; LRHT, the dietary addition of RPG was 1%, and the addition of TAU was 15 g/d; HRLT, the dietary addition of RPG was 3%, and the addition of TAU was 5 g/d; and HRHT, the dietary addition of RPG was 3%, and the addition of TAU was 15 g/d. In the “R” to the right of this line, “LR” refers to LRLT and LRHT, while “HR” refers to HRLT and HRHT; In the “T,” “LT” refers to LRLT and HRLT, while HT refers to LRHT and HRHT. ^3^“R” means the RPG; “T” means the TAU; and “R*T” means the interaction between RPG and TAU.

### 3.3 Changes of biochemical, antioxidant, immune and stress response indicators in plasma before and after transportation

We did not observe any changes in the concentrations (*P* > 0.05) of ALT, LAC, TG, and GLU in plasma before and after transport or among different treatments (Table 4). Compared with before transport, the concentration of TC, TP, LDH and CK in plasma of yaks with different treatments increased after transport (*P* < 0.01). The plasma TP concentration before transport was higher in HRHT than in LRLT (*p* = 0.04). The changes of antioxidant, immune and stress response indicators in plasma before and after transportation are provided in Tables 5–7. Transport increased plasma concentrations of MDA (*p* = 0.05), COR (*P* < 0.01) and LPS (*P* < 0.01), while decreased plasma IL-10 concentration (*P* < 0.01). Increasing TAU supplementation increased plasma GSH-PX activity before (*p* = 0.02) and after transportation (*p* = 0.03). The plasma MDA concentrations after transport was lower in HRHT than in LRLT (*p* = 0.03, 15.1 vs. 16.7 nmol/mL). The plasma IL-4 concentration after transport was higher in HRHT than in LRLT (*p* = 0.03). The plasma IL-10 concentration after transport was higher in LRHT and HRHT than in LRLT and HRLT (*p* = 0.02). In addition, increasing TAU supplementation decreased the plasma concentration of COR before transportation (*p* = 0.02) as well as LPS before (*p* = 0.05) and after transportation (*p* = 0.04). The plasma COR concentration after transport was lower in LRHT and HRHT than in LRLT (*p* = 0.03).

**Table 4:** Changes in plasma biochemical of yaks^1^ before and after transport under various treatments.

Items	Transport	Treatment^2^				SEM	*P*-value^3^			
		LRLT	LRHT	HRLT	HRHT		S	R	T	R*T
TC (mmol/L)	Before	1.84	2.04	1.94	1.77	0.12	< 0.01	0.36	0.88	0.17
	After	2.92	2.88	3.25	2.83	0.27		0.46	0.22	0.38
TP (g/L)	Before	47.8^b^	53.7^ab^	51.7^ab^	55.5^a^	2.58	< 0.01	0.16	0.02	0.04
	After	73.6	71.2	74.2	73.5	2.23		0.38	0.32	0.57
ALT (U/L)	Before	54.5	57.3	57.9	58.8	3.62	0.92	0.34	0.47	0.68
	After	56.2	54.6	57.8	60.2	3.74		0.17	0.88	0.49
LAC (U/L)	Before	63.9	62.4	61.1	62.7	2.59	0.36	0.49	0.99	0.75
	After	62.2	60.4	62.5	61.3	1.47		0.60	0.15	0.50
TG (mmol/L)	Before	0.13	0.16	0.14	0.13	0.02	0.87	0.45	0.39	0.35
	After	0.14	0.12	0.16	0.13	0.02		0.34	0.07	0.19
GLU (mmol/L)	Before	2.95	2.49	2.53	2.67	0.24	0.80	0.47	0.36	0.22
	After	2.86	2.61	2.51	2.77	0.17		0.44	0.96	0.17
LDH (U/L)	Before	875.2	924.7	894.3	853.1	42.8	< 0.01	0.39	0.89	0.41
	After	1305.8	1236.7	1275.3	1228.3	41.9		0.53	0.08	0.25
CK (U/L)	Before	129.5	132.0	126.9	123.2	12.0	< 0.01	0.50	0.95	0.90
	After	392.9	374.2	362.7	320.3	35.1		0.10	0.24	0.23

^1^Values are least squares means of eight replicate yaks (*n* = 8). ^2^LRLT, the dietary addition of RPG was 1%, and the addition of TAU was 5 g/d; LRHT, the dietary addition of RPG was 1%, and the addition of TAU was 15 g/d; HRLT, the dietary addition of RPG was 3%, and the addition of TAU was 5 g/d; and HRHT, the dietary addition of RPG was 3%, and the addition of TAU was 15 g/d. ^3^“S” means the transportation; “R” means the RPG; “T” means the TAU; and “R*T” means the interaction between RPG and TAU. ^a, b^Means within a row with different superscripts differ significantly (*p* ≤ 0.05).

**Table 5:** Changes in plasma antioxidant capacity of yaks^1^ before and after transport under various treatments.

Items	Transport	Treatment^2^				SEM	*P*-value^3^			
		LRLT	LRHT	HRLT	HRHT		S	R	T	R*T
CAT (U/mL)	Before	17.5	17.1	18.0	17.8	0.76	0.26	0.27	0.56	0.67
	After	18.2	18.0	17.8	18.3	0.92		0.91	0.77	0.96
SOD (U/mL)	Before	39.6	40.3	40.5	40.7	1.43	0.27	0.46	0.66	0.86
	After	39.8	41.1	41.3	43.3	2.48		0.30	0.35	0.59
GSH-PX (U/mL)	Before	434.4	469.0	447.9	472.4	16.8	0.19	0.53	0.02	0.12
	After	426.2	454.2	440.0	461.6	15.2		0.38	0.03	0.14
T-AOC (μmol/mL)	Before	2.81	2.83	2.90	2.84	0.18	0.35	0.67	0.89	0.96
	After	2.64	2.77	2.84	2.74	0.25		0.62	0.93	0.88
MDA (nmol/mL)	Before	16.0	14.9	15.6	14.7	0.53	0.05	0.75	0.03	0.16
	After	16.7^a^	15.8^ab^	16.3^ab^	15.1^b^	0.48		0.17	0.01	0.03

^1^Values are least squares means of eight replicate yaks (*n* = 8). ^2^LRLT, the dietary addition of RPG was 1%, and the addition of TAU was 5 g/d; LRHT, the dietary addition of RPG was 1%, and the addition of TAU was 15 g/d; HRLT, the dietary addition of RPG was 3%, and the addition of TAU was 5 g/d; and HRHT, the dietary addition of RPG was 3%, and the addition of TAU was 15 g/d. ^3^“S” means the transportation; “R” means the RPG; “T” means the TAU; and “R*T” means the interaction between RPG and TAU. ^a,b^Means within a row with different superscripts differ significantly (*p* ≤ 0.05).

**Table 6:** Changes in plasma immune index of yaks^1^ before and after transport under various treatments.

Items	Transport	Treatment^2^				SEM	*P*-value^3^			
		LRLT	LRHT	HRLT	HRHT		S	R	T	R*T
IL-1β (ng/L)	Before	54.9	55.9	56.1	56.1	1.59	0.08	0.50	0.64	0.85
	After	55.7	57.4	57.7	56.9	0.99		0.34	0.58	0.25
IL-4 (ng/L)	Before	89.4	94.9	94.3	95.7	2.05	0.11	0.22	0.13	0.20
	After	90.5^b^	91.6^ab^	91.0^ab^	93.5^a^	0.94		0.14	0.02	0.03
IL-6 (ng/L)	Before	79.8	81.9	81.7	80.1	1.99	0.23	0.96	0.81	0.63
	After	81.9	82.5	82.1	81.7	1.97		0.81	0.96	0.98
IL-10 (ng/L)	Before	44.1	44.6	43.6	45.8	1.32	< 0.01	0.72	0.15	0.41
	After	41.5^b^	43.0^a^	41.2^b^	43.3^a^	0.72		0.99	0.01	0.02
TNF-α (ng/L)	Before	271.3	262.4	274.7	258.2	11.2	0.28	0.97	0.10	0.45
	After	284.8	266.7	280.9	259.9	11.4		0.56	0.02	0.14

^1^Values are least squares means of eight replicate yaks (*n* = 8). ^2^LRLT, the dietary addition of RPG was 1%, and the addition of TAU was 5 g/d; LRHT, the dietary addition of RPG was 1%, and the addition of TAU was 15 g/d; HRLT, the dietary addition of RPG was 3%, and the addition of TAU was 5 g/d; and HRHT, the dietary addition of RPG was 3%, and the addition of TAU was 15 g/d. ^3^“S” means the transportation; “R” means the RPG; “T” means the TAU; and “R*T” means the interaction between RPG and TAU. ^a,b^Means within a row with different superscripts differ significantly (*p* ≤ 0.05).

**Table 7:** Changes in plasma stress response indicators of yaks^1^ before and after transport under various treatments.

Items	Transport	Treatment^2^				SEM	*P*-value^3^			
		LRLT	LRHT	HRLT	HRHT		S	R	T	R*T
COR (ng/mL)	Before	185.3	175.9	183.8	168.2	7.30	< 0.01	0.43	0.02	0.11
	After	266.8^a^	237.8^b^	248.4^ab^	234.9^b^	10.1		0.23	0.01	0.03
LPS (EU/L)	Before	385.7	366.1	380.7	352.5	15.8	< 0.01	0.45	0.05	0.18
	After	473.2	448.3	470.3	431.4	19.6		0.31	0.04	0.19

^1^Values are least squares means of eight replicate yaks (*n* = 8). ^2^LRLT, the dietary addition of RPG was 1%, and the addition of TAU was 5 g/d; LRHT, the dietary addition of RPG was 1%, and the addition of TAU was 15 g/d; HRLT, the dietary addition of RPG was 3%, and the addition of TAU was 5 g/d; and HRHT, the dietary addition of RPG was 3%, and the addition of TAU was 15 g/d. ^3^“S” means the transportation; “R” means the RPG; “T” means the TAU; and “R*T” means the interaction between RPG and TAU. ^a,b^Means within a row with different superscripts differ significantly (*p* ≤ 0.05).

### 3.4 Plasma antioxidant in yaks after transportation

We found no significant changes (*p* > 0.05) in the activities of CAT and SOD, T-AOC as well as MDA concentrations in plasma on days 10, and 30 post-transport across treatments (Table 8). The TAU (*p* = 0.03) and the interaction between RPG and TAU (*p* = 0.04) had significant effects on plasma GSH-PX activities at day 10 d after transport, which was lower in HRHT than in LRLT.

**Table 8:** Effects of RPG and TAU levels in the diet on the plasma antioxidant capacity of yaks^1^ after transportation.

Items	Treatment^2^				SEM	R		T		*P*-value^3^		
	LRLT	LRHT	HRLT	HRHT		LR	HR	LT	HT	R	T	R*T
CAT (U/mL)												
10 d	17.0	17.6	17.1	17.1	1.15	17.3	17.1	17.1	17.3	0.75	0.72	0.94
30 d	16.9	17.5	16.8	17.0	0.64	17.2	16.9	16.8	17.3	0.52	0.35	0.71
SOD (U/mL)												
10 d	39.6	42.6	40.2	41.8	2.03	41.1	41.0	39.9	42.2	0.98	0.11	0.44
30 d	40.9	42.2	42.3	43.2	1.85	41.6	42.7	41.6	42.7	0.37	0.41	0.69
GSH-PX (IU/L)												
10 d	422.5^b^	452.2^ab^	445.8^ab^	463.1^a^	13.1	437.5	454.5	434.1	457.8	0.12	0.03	0.04
30 d	423.9	427.1	441.8	450.1	16.1	425.0	445.9	432.4	438.6	0.07	0.60	0.33
T-AOC (μmol/mL)												
10 d	2.68	2.82	2.65	2.57	0.24	2.75	2.61	2.66	2.70	0.39	0.83	0.76
30 d	2.67	2.77	2.67	2.53	0.11	2.72	2.60	2.67	2.65	0.15	0.83	0.25
MDA (nmol/mL)												
10 d	16.0	16.5	16.3	15.6	0.56	16.3	15.9	16.2	16.0	0.44	0.67	0.42
30 d	15.4	15.9	16.0	15.6	0.64	15.6	15.8	15.7	15.7	0.72	0.90	0.81

^1^Values are least squares means of eight replicate yaks (*n* = 8). ^2^LRLT, the dietary addition of RPG was 1%, and the addition of TAU was 5 g/d; LRHT, the dietary addition of RPG was 1%, and the addition of TAU was 15 g/d; HRLT, the dietary addition of RPG was 3%, and the addition of TAU was 5 g/d; and HRHT, the dietary addition of RPG was 3%, and the addition of TAU was 15 g/d. In the “R” to the right of this line, “LR” refers to LRLT and LRHT, while “HR” refers to HRLT and HRHT; In the “T,” “LT” refers to LRLT and HRLT, while HT refers to LRHT and HRHT. ^3^“R” means the RPG; “T” means the TAU; and “R*T” means the interaction between RPG and TAU. ^a,b^Means within a row with different superscripts differ significantly (*p* ≤ 0.05).

### 3.5 Plasma immune in yaks after transportation

Table 9 shows the effects of dietary RPG level and TAU supplementation on the plasma immune index of yak after transportation. Increasing TAU supplementation decreased plasma IL-1β concentrations at day 30 after transport (*p* = 0.02), while increased plasma IL-4 concentrations at day 10 after transport (*p* = 0.02). Besides that, plasma immune index did not differ at all treatments (*P* > 0.05).

**Table 9:** Effects of RPG and TAU levels in the diet on the plasma immune index of yaks^1^ after transportation.

Items	Treatment^2^				SEM	R		T		*P*-value^3^		
	LRLT	LRHT	HRLT	HRHT		LR	HR	LT	HT	R	T	R*T
IL-1β (ng/L)												
10 d	55.5	55.9	57.0	56.4	1.04	55.7	56.7	56.3	56.1	0.16	0.88	0.52
30 d	56.4	54.5	56.3	55.3	0.77	55.5	55.8	56.3	54.9	0.62	0.02	0.09
IL-4 (ng/L)												
10 d	90.7	91.5	89.2	92.7	1.13	91.1	91.0	90.0	92.1	0.88	0.02	0.07
30 d	92.7	93.8	90.9	92.7	1.71	93.2	91.8	91.8	93.3	0.23	0.22	0.41
IL-6 (ng/L)												
10 d	82.4	82.0	82.7	81.0	2.13	82.2	81.9	82.6	81.5	0.91	0.71	0.88
30 d	76.5	75.5	76.4	75.3	2.67	76.0	75.8	76.4	75.4	0.96	0.74	0.79
IL-10 (ng/L)												
10 d	41.7^b^	43.2^ab^	42.3^b^	44.9^a^	0.80	42.4	43.6	41.9	44.0	0.13	< 0.01	0.01
30 d	42.8	44.1	43.3	44.3	1.17	43.4	43.8	43.1	44.2	0.76	0.35	0.82
TNF-α (ng/L)												
10 d	282.1	279.6	277.2	273.9	13.7	280.8	275.6	279.7	276.7	0.57	0.76	0.94
30 d	281.2	272.1	272.7	265.2	8.78	276.6	269.0	276.9	268.7	0.23	0.20	0.38

^1^Values are least squares means of eight replicate yaks (*n* = 8). ^2^LRLT, the dietary addition of RPG was 1%, and the addition of TAU was 5 g/d; LRHT, the dietary addition of RPG was 1%, and the addition of TAU was 15 g/d; HRLT, the dietary addition of RPG was 3%, and the addition of TAU was 5 g/d; and HRHT, the dietary addition of RPG was 3%, and the addition of TAU was 15 g/d. In the “R” to the right of this line, “LR” refers to LRLT and LRHT, while “HR” refers to HRLT and HRHT; In the “T,” “LT” refers to LRLT and HRLT, while HT refers to LRHT and HRHT. ^3^“R” means the RPG; “T” means the TAU; and “R*T” means the interaction between RPG and TAU. ^a,b^Means within a row with different superscripts differ significantly (*p* ≤ 0.05).

### 3.6 Plasma stress response indicators in yaks after transportation

As shown in Table 10, the RPG and TAU did not change the plasma LPS concentration of yaks after transport (*P* > 0.05). However, the plasma COR concentration in yaks decreased at 10 (*p* = 0.03) and 30 (*p* = 0.05) days after transport with the increase of TAU addition.

**Table 10:** Effects of RPG and TAU levels in the diet on the plasma stress response indicators of yaks^1^ after transportation.

Items	Treatment^2^				SEM	R		T		*P*-value^3^		
	LRLT	LRHT	HRLT	HRHT		LR	HR	LT	HT	R	T	R*T
COR (ng/mL)												
10 d	218.9	208.7	216.0	200.8	7.96	213.8	208.4	217.4	204.7	0.39	0.03	0.15
30 d	191.5	182.1	189.9	181.3	6.35	186.8	185.7	190.7	181.7	0.82	0.05	0.29
LPS (EU/L)												
10 d	408.7	367.9	384.9	357.1	21.8	388.3	371.0	396.8	362.5	0.31	0.09	0.14
30 d	361.7	346.7	364.3	330.7	19.4	354.2	347.5	363.0	338.7	0.65	0.08	0.32

^1^Values are least squares means of eight replicate yaks (*n* = 8). ^2^LRLT, the dietary addition of RPG was 1%, and the addition of TAU was 5 g/d; LRHT, the dietary addition of RPG was 1%, and the addition of TAU was 15 g/d; HRLT, the dietary addition of RPG was 3%, and the addition of TAU was 5 g/d; and HRHT, the dietary addition of RPG was 3%, and the addition of TAU was 15 g/d. In the “R” to the right of this line, “LR” refers to LRLT and LRHT, while “HR” refers to HRLT and HRHT; In the “T,” “LT” refers to LRLT and HRLT, while HT refers to LRHT and HRHT. ^3^“R” means the RPG; “T” means the TAU; and “R*T” means the interaction between RPG and TAU.

## 4 Discussion

Harsh conditions during transportation, like noise and overcrowding, can induce oxidative stress in animals, resulting in weight loss. Lendrawati et al. (17) reported that transport reduces the weight of sheep, and the weight loss increases with the longer the transport time. González et al. (18) reported that while rearing patterns, age, transportation time, and truck facilities all affect the weight of cattle transported long distances, these tests all show a decrease in weight. In our study, all yaks experienced weight loss after 9 h of long-distance transport, and even supplementation with high levels of RPG and TAU could not reverse this outcome. However, yaks fed high supplemental TAU had lower weight loss (8.42 vs. 11.9 kg) and lower weight loss percent (4.83 vs. 6.87%), which was closely related to the biological function of TAU. Mice with inadequate TAU intake reportedly exhibited low body weight, slower responses to stimuli, and exercise intolerance, indicating that TAU was crucial for maintaining body weight and muscle endurance (19). Kim et al. (20) demonstrated the fatigue resistance of TAU following acute exercise, which were attributed to increased oxygen uptake and enhanced lipid oxidation in a mouse model. Taurine is also a promoter of energy metabolic balance (13) and may help increase energy supply of yak during transport. In addition, feeding TAU can improve the performance of animals (16, 21). However, we found that the growth performance of yaks within 30 days after transport did not change due to the increase in TAU and RPG. One possible reason is that this study focused more on the dosage of RPG and TAU rather than whether they were added, which may limit the comparison of this work with other studies and will be refined in future work.

Plasma TP is clinically significant for assessing liver synthesis function, nutritional status, and water balance. Mufarrej et al. (22) observed that transport increased the levels of TP and albumin in plasma of lambs. In our study, long-term transport may promote the fluid loss in yaks, causing blood to become concentrated and plasma TP levels to rise. Plasma TC concentration is commonly used to evaluate lipid metabolism and diagnose certain diseases (23, 24). We observed an increase in plasma TC concentration post-transport, likely due to the damage and dysfunction of cell membranes caused by lipid peroxidation, which affects the metabolism of lipoproteins and thus indirectly affects the TC in plasma. Plasma levels of LDH and CK are considered biomarkers of muscle injury (25, 26). Fang et al. (27) reported that transport induced an increase in plasma LDH concentration and changes in other blood indicators, suggesting the occurrence of oxidative stress. Alcalde et al. (28) also reported that road transportation, irrespective of the duration, consistently increased plasma CK levels in suckling goat kids, suggesting significant stress and muscle damage. Similarly, we observed elevated levels of LDH and CK in yak plasma post-transport, indicating potential health risks for yaks.

Stress during transport negatively affects the antioxidant system in animals, resulting in reduced antioxidant enzyme activity, which hinders the effective removal of free radicals and peroxide products from the body (29, 30). MDA is the end product of lipid peroxidation, shows a positive correlation between its plasma concentration and the severity of oxidative stress (31). Transport leads to increased plasma MDA concentration, indicating the onset and progression of oxidative stress (32, 33), which is also supported by our study findings. We observed that increasing TAU levels reduced plasma MDA concentration in yaks after transport, mitigating transport stress. Han et al. (16) found that TAU alleviates oxidative damage by increasing gene expression of NRF2, GPX and HO-1. Seidel et al. (34) also found that TAU reduces cellular stress levels, possibly by upregulating iron-related proteins like iron storage protein, thereby inhibiting lipid peroxidation. Therefore, we speculated that increasing TAU levels alleviated transport stress in yaks by inhibiting lipid peroxidation, and the specific mechanism needs to be further explored. GSH-PX is crucial for cellular antioxidant defense as it eliminates ROS from the body. Han et al. (35) indicated that TAU supplementation increased the glutathione contents and the GSH-PX activity, alleviating the oxidative stress induced by LPS. In our study, at 0 and 10 days after transport, increased TAU supplementation elevated plasma GSH-PX activity, implying an enhanced antioxidant capacity in yaks. It is to be noted that the plasma antioxidant capacity differences among treatments diminished over time after transport, possibly because the antioxidant function of TAU was activated in majority cases when exposed to oxidative stress risk.

During oxidative stress, the body generates a surplus of ROS, which activate the inflammasome and trigger the release of numerous pro-inflammatory cytokines, leading to cellular damage (36). Transport increased the concentration of proinflammatory cytokines like TNF-*α* and IL-1β in animal plasma (37, 38). However, in our study, transport did not change the concentration of IL-1β, IL-6 and TNF-α, while decreased the concentration of anti-inflammatory factors like IL-10, which also means that the health status of yaks may be potentially damaged. In addition, we found that increasing TAU supplementation decreased plasma IL-1β concentrations at 30 day after transport and TNF-*α* concentrations at 0 day after transport. TAU has been demonstrated to mitigates the adverse effects of LPS by dampening pro-inflammatory responses and oxidative stress (39). Zheng et al. (40) reported that TAU deficiency aggravated the DSS induced inflammatory model in mice, while TAU supplementation reduced proinflammatory factors levels like TNF-α and IL-6 by inhibiting the TLR4/NF-κB pathway. Han et al. (35) also indicated that TAU supplementation decreased the expression of IL-1β, TNF-α, and IL-6 genes, thereby resisting the LPS-induced inflammatory response in broilers. The release of pro-inflammatory cytokines accompanies the onset of inflammation, and over time, the pathogen is cleared by the body, and anti-inflammatory cytokines then come into play to repair the damaged tissue (41). TAU has a positive effect on the formation of the immune system of body (42). Zhou et al. (43) reported that TAU increases IL-10 concentration in weaned piglets and enhances immunity against weaning stress and inflammation. Elevated plasma concentrations of IL-4 and IL-10 in yaks were observed following transport when RPG and TAU were combined at high levels, indicating enhanced immunity, yet these levels stabilized after 30 days of feeding. It is likely that TAU exerts an anti-inflammatory effect during transport stress occurs, but the influence wanes when transport stress diminishes or even disappears.

The COR is an important glucocorticoid, and its concentrations serve as a crucial index for assessing stress responses, with their dynamic changes being closely tied to stress progression (44). Lee et al. (45) reported that road transport increased the plasma COR concentration of dairy cows. In our study, the concentrations of COR and LPS in yak plasma increased after transport, indicating the occurrence of transport stress. We also observed that the increase of TAU supplementation decreased the COR concentrations in yaks throughout the experiment, which suggested that TAU could mitigate transport stress in yaks, with a lasting beneficial effect (30 d). LPS stimulates the immune system, thereby triggering inflammation. He et al. (46) reported that transport impaired morphology and tissue in jejunum, induced stress responses, and elevated LPS concentrations. In our study, the LPS concentration of yaks decreased before and after transport with the increase of TAU addition, which was beneficial to the health of yaks after transport.

## 5 Conclusion

Transport induces oxidative stress in yaks, which can lead to reduced body weight and health impairment. TAU mitigates the adverse effects of transport on yak weight. Increasing TAU levels enhanced antioxidant capacity and immunity of yaks after transport, alleviating their oxidative stress, but this beneficial effect was weakened with time after transport. RPG had less effect on transport stress than TAU. Considering both cost and efficacy, the recommended dietary inclusion of RPG for yaks is 1%, with a TAU supplement of 15 g/d to mitigate transport stress. This work presents an effective strategy for mitigating transport stress, which is of great significance for off-site fattening of grazing yaks in the cold season.

## Data Availability

The raw data supporting the conclusions of this article will be made available by the authors, without undue reservation.
